# Modulation of Somatosensory Alpha Rhythm by Transcranial Alternating Current Stimulation at Mu-Frequency

**DOI:** 10.3389/fnhum.2017.00432

**Published:** 2017-08-25

**Authors:** Christopher Gundlach, Matthias M. Müller, Till Nierhaus, Arno Villringer, Bernhard Sehm

**Affiliations:** ^1^Department for Neurology, Max Planck Institute for Human Cognitive and Brain Sciences Leipzig, Germany; ^2^Institute of Psychology, University of Leipzig Leipzig, Germany; ^3^Center for Cognitive Neuroscience Berlin, Freie Universität Berlin, Germany; ^4^MindBrainBody Institute, Berlin School of Mind and Brain, Charité Universitätsmedizin Berlin, Humboldt University of Berlin Berlin, Germany; ^5^Clinic for Cognitive Neurology, University of Leipzig Leipzig, Germany

**Keywords:** transcranial alternating current stimulation, tACS, mu-alpha, brain oscillations, electroencephalogram, EEG, somatosensory cortex

## Abstract

**Introduction**: Transcranial alternating current stimulation (tACS) is emerging as an interventional tool to modulate different functions of the brain, potentially by interacting with intrinsic ongoing neuronal oscillations. Functionally different intrinsic alpha oscillations are found throughout the cortex. Yet it remains unclear whether tACS is capable of specifically modulating the somatosensory mu-rhythm in amplitude.

**Objectives**: We used tACS to modulate mu-alpha oscillations in amplitude. When compared to sham stimulation we expected a modulation of mu-alpha oscillations but not visual alpha oscillations by tACS.

**Methods**: Individual mu-alpha frequencies were determined in 25 participants. Subsequently, blocks of tACS with individual mu-alpha frequency and sham stimulation were applied over primary somatosensory cortex (SI). Electroencephalogram (EEG) was recorded before and after either stimulation or sham. Modulations of mu-alpha and, for control, visual alpha amplitudes were then compared between tACS and sham.

**Results**: Somatosensory mu-alpha oscillations decreased in amplitude after tACS was applied at participants’ individual mu-alpha frequency. No changes in amplitude were observed for sham stimulation. Furthermore, visual alpha oscillations were not affected by tACS or sham, respectively.

**Conclusion**: Our results demonstrate the capability of tACS to specifically modulate the targeted somatosensory mu-rhythm when the tACS frequency is tuned to the individual endogenous rhythm and applied over somatosensory areas. Our results are in contrast to previously reported amplitude increases of visual alpha oscillations induced by tACS applied over visual cortex. Our results may point to a specific interaction between our stimulation protocol and the functional architecture of the somatosensory system.

## Introduction

Major functional modes of the human brain rely on neuronal oscillatory activity across different temporal and spatial scales (Engel et al., 2001; Buzsáki and Draguhn, 2004; Jensen and Mazaheri, 2010). Modulations of oscillatory activity are associated with various cognitive as well as perception-related processes (Engel and Singer, 2001; Engel et al., 2001; Koepsell et al., 2010; Wang, 2010; Vanrullen and Dubois, 2011). In the somatosensory system the predominant neuronal oscillation is the mu- or rolandic rhythm with frequency peaks in the alpha and beta range (Pfurtscheller and Lopes da Silva, 1999). Dynamics of this brain rhythm in amplitude and phase have been shown to be related to different aspects of somatosensory processing such as perception of near-threshold stimuli and related attentional processes (Linkenkaer-Hansen et al., 2004; Palva et al., 2005; Schubert et al., 2009; Jones et al., 2010; van Ede et al., 2011). More generally, functionally different alpha oscillations are found throughout the cortex (Hari et al., 1997; Niedermeyer, 1997; Pineda, 2005; Weisz et al., 2011; Haegens et al., 2015) and seem to play a pivotal role in the modulation and orchestration of information flow across sensory domains (Klimesch et al., 2007; Jensen and Mazaheri, 2010; Foxe and Snyder, 2011; Mathewson et al., 2011).

Non-invasive brain stimulation methods such as transcranial alternating current stimulation (tACS) may offer a possibility to modulate neuronal oscillatory activity (for a review, see Herrmann et al., 2013) and human brain function in different modalities such as vision (Kanai et al., 2008; Laczó et al., 2012; Brignani et al., 2013; Helfrich et al., 2014a,b; Cabral-Calderin et al., 2015; Vossen et al., 2015; Kasten et al., 2016; Vosskuhl et al., 2016), motor function (Pogosyan et al., 2009; Feurra et al., 2011a; Schutter and Hortensius, 2011; Joundi et al., 2012; Brittain et al., 2013; Wach et al., 2013; Krause et al., 2014; Brinkman et al., 2016; Lustenberger et al., 2016; Moisa et al., 2016), audition (Neuling et al., 2012; Riecke et al., 2015; Heimrath et al., 2016; Riecke, 2016; Rufener et al., 2016), somatosensation (Feurra et al., 2011b; Gundlach et al., 2016), and higher cognitive functions such as decision making, risk taking behavior, creativity, fluid intelligence, mental rotation or self-aware dreaming (Sela et al., 2012; Herrmann et al., 2013; Voss et al., 2014; Lustenberger et al., 2015; Santarnecchi et al., 2016; Kasten and Herrmann, 2017). In addition there is preliminary evidence that alternating current stimulation might be effective to support recovery of function in patients with stroke (Fedorov et al., 2010; Naros and Gharabaghi, 2017) or optic neuropathy (Sabel et al., 2011; Schmidt et al., 2013).

However, it still remains largely elusive, what mechanisms are mediating these effects in the human brain. Evidence from animal and modeling studies suggest, that weak alternating electric fields have an influence on modulating spiking patterns of neurons by an interaction between ongoing oscillatory activity and applied oscillations (Deans et al., 2007; Fröhlich and McCormick, 2010; Ozen et al., 2010; Reato et al., 2010) and that these online effects are likely due to entrainment of ongoing oscillations by tACS (Herrmann et al., 2013; Reato et al., 2013). In humans, the application of tACS in the alpha range also modulated ongoing visual alpha oscillations in amplitude during and after stimulation (Zaehle et al., 2010; Neuling et al., 2013; Helfrich et al., 2014a,b; Strüber et al., 2015; Vossen et al., 2015; Kasten et al., 2016). However, there is evidence that the offline effects that were found after the stimulation may be distinct from online entrainment effects (Zaehle et al., 2010; Strüber et al., 2015; Veniero et al., 2015; Vossen et al., 2015).

Additionally a modulation of ongoing oscillations induced by tACS has so far only been shown for alpha oscillations in the visual cortex. It is as yet unknown whether neuronal oscillations originating from different cortices, such as the somatosensory mu-alpha rhythm, might also be modulated by tACS, and whether potential modulations are specific to this rhythm. The aim of the present study was to investigate the effects of tACS tuned to participants’ individual mu-alpha frequency (mu-tACS) on ongoing somatosensory mu-alpha oscillations in human electroencephalogram (EEG) recordings. For this purpose mu-tACS was applied bilaterally over primary somatosensory cortices (SI) and compared to sham stimulation. Modulations of mu-alpha oscillations after the end of the stimulation as compared to before stimulation were then compared between tACS and sham-stimulation. We hypothesized, that tACS tuned to individual somatosensory alpha frequency induces changes in mu-alpha amplitudes. Additionally, we expected specific changes for somatosensory mu-alpha oscillations and no changes for visual alpha.

## Materials and Methods

### Participants

Twenty-five healthy participants (12 female, mean age 27, *SD* = 2.97) participated in a single-blinded combined EEG and tACS experiment. One participant reported to have fallen asleep and was discarded, another was discarded from analysis due to artifacts related to electrical bridging between C3/C4 electrodes and stimulation electrodes. Hence 23 subjects (11 female, mean age 26.96, *SD* = 3.09) entered the analysis. All participants were right-handed according to the Oldfield questionnaire for the assessment of handedness (Oldfield, 1971). Prior to the study, participants gave written informed consent to participate in the experiment and underwent a neurological examination. Participants were not taking any medication. The study was designed and conducted according to the declaration of Helsinki and was approved by the ethics committee of the University of Leipzig.

### Transcranial Alternating Current Stimulation (tACS)

Electric stimulation was delivered with a battery-operated stimulator system (ELDITH, Neuroconn, Ilmenau, Germany) via two rubber electrodes (40 × 40 mm) placed over CP3 and CP4 underneath an EEG elastic cap. The impedance was kept below 10 kΩ by applying electrode gel (Ten20, D.O. Weaver, Aurora, CO, USA) between skin and electrode. The stimulation intensity was kept at 1 mA (peak to peak) resulting in a maximum current density of 62.5 μA/cm^2^ under the stimulation electrodes. The study consisted of one tACS block (duration of 5 min) and a sham stimulation block. The order of the blocks were counterbalanced across subjects. For the *verum* stimulation block, participants’ individual mu-alpha frequency was used as determined in a pre-experiment (see below, “Experimental design” Section). For sham stimulation, stimulation site was kept constant, but we used a fixed frequency of 10 Hz and a duration of 30 s to mimic transient tingling sensations associated with the onset of real stimulation (Gandiga et al., 2006). For each block, the first 10 and the last 2 s were ramped up and down.

### EEG

**Figure 1:**
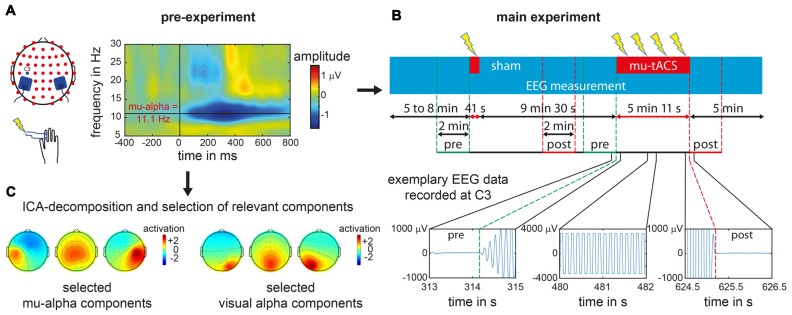
Experimental procedure for an exemplary single subject. **(A)** In the pre-experiment suprathreshold electric stimuli were applied at right index finger. Electroencephalogram (EEG) data recorded over left primary somatosensory cortex (SI) at electrode C3 was analyzed with respect to the stimulus locked event related desynchronization (ERD) as represented in the time frequency analysis plot for a single subject. From the ERD pattern the individual mu-alpha frequency was extracted as the maximum post-stimulus decrease in amplitude in the alpha-band (8–14 Hz) in the time window of 200–600 ms. **(B)** During the main experiment one sham block and one transcranial alternating current stimulation (tACS) block with participant’s individual mu-alpha frequency (mu-tACS) were applied while the EEG was recorded. Later data analysis was restricted to 2 min pre- and post-stimulation as tACS led to strong artifacts during stimulation with data clipping due to amplifier saturation (as illustrated for EEG signals recorded at electrode C3 for a single subject during the stimulation and its onset and offset). **(C)** For oscillation specific analysis of the data, pre-experimental EEG data was decomposed via an Independent Component Analysis (ICA) and mu-alpha related as well as visual alpha related components were subsequently selected (exemplary component sets for a single subject are represented).

EEG was recorded using a 52 passive electrodes setup mounted in an elastic cap based on the standard international 10–10 system (American Electroencephalographic Society, 1994), at a sampling rate of 2500 Hz using a BrainAmp amplifier (Brain Products, Munich, Germany). Due to the positioning of the tACS electrodes, the electrodes CP3, CP5, P3, P5, CP4, CP6, P4 and P6 were omitted (see Figure 1A). EEG was recorded with left mastoid as a reference and later re-referenced to the average reference. For later offline analysis, EEGLAB (Delorme and Makeig, 2004) and custom Matlab scripts (The MathWorks, Natick, MA, USA) were used, while statistical analyses were performed using R (R Core Team, 2016).

### Experimental Design

Participants were seated in a comfortable chair inside a shielded EEG chamber while engaged in two parts of the experiment: pre-experiment and main experiment.

#### Pre-Experiment

The pre-experiment was conducted to determine each participant’s individual mu-alpha frequency. Therefore, each participant performed a passive somatosensory experiment with simultaneous EEG recording. While participants were fixating a centrally presented cross on a screen, 150 electric supra-threshold stimuli were applied to the right index finger via two Velcro ring-electrodes using a DS7 isolated bipolar constant current stimulator (Digitimer Ltd, Welwyn Garden City, Hertfordshire, UK). Intensity levels were in advance set to levels that were clearly perceivable but not yet uncomfortable. The electric stimuli were delivered with a mean interstimulus interval of 2 s and a maximum jitter of 1 s. Immediately after the pre-experiment, the EEG data were analyzed to extract the Event-Related Desynchronization (ERD) of the mu rhythm to the presented stimuli (Pfurtscheller et al., 1997; Pfurtscheller and Lopes da Silva, 1999). The frequency with the maximum ERD was identified (see “Data analysis” Section below) to serve as the individual target stimulation frequency for the subsequent experiment (see also Gundlach et al., 2016; see Figure 1A).

#### Main Experiment

The main experiment had a total duration of around 29 min. During the experiment, participants were seated in a comfortable chair with the EEG and tACS electrodes mounted on the scalp. In order to prevent stronger decreases in vigilance, a soundless documentary film was presented on a screen. The starting point of the documentary was randomized across participants to minimize any potential systematic impact of the documentary.

One block of verum tACS and one block of sham stimulation were applied during ongoing EEG recording. The sequence of both blocks was counterbalanced across subjects. The first block started randomly after 5, 6 or 7 min and between both blocks was a stimulation free interval of 5 min.

Before and after the main experiment, participants were asked to rate their current level of attention, tiredness, and pain on a 10-level visual analog scale. After the experiment, participants had to report: (i) whether they felt the stimulation, and if yes, how sure they were about this (10-level visual analog scale); (ii) whether they felt differences in stimulation, and if yes, how sure they were about this (10-level visual analog scale).

### Data Analysis

#### Pre-Experiment

In order to determine participants’ individual mu-alpha frequency, data of the pre-experiment recorded at electrode C3 were cut into epochs according to the trigger of the presented stimulus stretching from 1500 ms pre stimulus to 1500 ms post stimulus. For each trial, signals measured at electrode C3 were wavelet-transformed from 5 Hz to 35 Hz with 0.1 Hz increments using five cycle long wavelets in order to analyze the amplitude time course of various frequencies. A baseline time window from 600 ms to 300 ms pre stimulus was subtracted and time courses were averaged across trials to reveal stimulus related changes of neural oscillations. Amplitude values from 200 ms to 600 ms post stimulus were then averaged for each frequency. Within the alpha-band (8–14 Hz), the frequency with the maximum ERD (Pfurtscheller et al., 1997; Pfurtscheller and Lopes da Silva, 1999) i.e., the maximum amplitude difference between pre- and post-stimulus window, was extracted and served as our target stimulation frequency (mu-alpha, see Figure 1A).

#### Main Experiment

In order to examine effects of tACS on somatosensory mu-alpha oscillations, we compared participants’ individual mu-alpha amplitudes 2 min before (pre-stimulation) and after each stimulation block (post-stimulation). Analysis of EEG data recorded during the stimulation period was not possible due to saturation of the EEG amplifier by the tACS-induced signals (see Figure 1B). For the sham stimulation block (duration of only 30 s plus 12 s for ramping up and down the intensity), a time window comparable to that of the verum stimulation was chosen, i.e., the post-stimulation window started 5 min and 12 s after the beginning of the sham-stimulation.

EEG data was recalculated to average reference and cut into 120 segments with a duration of 1 s each. Noisy segments with amplitudes exceeding 100 μV were discarded (1.67% of all segments). Amplitude spectra were then calculated via a fast Fourier transform and averaged for each time window. Mean amplitude values of mu-alpha oscillations (mu-alpha peak frequency ±1 Hz, to compensate for fluctuations in peak frequency; Haegens et al., 2014) were then extracted at electrodes C3 and C4 (located over SI). Post-stimulation amplitude values were then normalized to pre-stimulation amplitude values to control for interindividual variance in mu-alpha amplitude. Potential systematic changes of amplitude values for the verum and sham stimulation were tested with *t*-tests against 0. Differences between tACS and sham were tested with a paired *t*-test. As a control measure potential baseline differences in the pre-stimulation amplitude values between tACS and sham block were tested with a paired *t*-test.

In a second step this analysis was repeated for separated somatosensory mu-alpha and visual alpha oscillations in order to examine the functional specificity of the tACS application. In general, signals measured at each EEG electrode represent a mixture of neural activity from different sources due to volume conduction processes. Additionally anatomically different sources of functionally diverging alpha oscillations haven been found in the cortex (Hari et al., 1997; Niedermeyer, 1997; Pineda, 2005; Weisz et al., 2011; Haegens et al., 2015). Alpha oscillations measured at each channel may therefore stem from different neural sources. In order to differentiate between sources of neural oscillations, we used an Independent Component Analysis (ICA) decomposition (Makeig et al., 1997) of the pre-experimental data set for each subject, using the Infomax approach implemented in EEGLAB (Delorme and Makeig, 2004). This resulted in a decomposition of the data into 52 maximally different components. First, only components that were related to somatosensory mu-alpha oscillations were selected based on criteria that were applied previously (see Reinacher et al., 2009; Freyer et al., 2012; Nierhaus et al., 2015; Forschack et al., 2017): they (i) had a central to left-lateralized topography; (ii) had amplitude peaks in the alpha and beta frequency bands; and (iii) showed a decrease in alpha and beta amplitude after the presentation of electric stimuli (Pfurtscheller et al., 1997; Pfurtscheller and Lopes da Silva, 1999). A median four components were selected for each subject (range from 1 to 8) and used as spatial filters for the back-projection of the EEG data of the main experiment, thus restricting our analysis to mu-oscillatory activity and simultaneously minimizing contamination by other alpha rhythms, such as visual alpha (see Figure 1C). For this back-projected EEG data, the same analysis as described above was done to test for systematic amplitude modulations of somatosensory mu-alpha oscillations by tACS as compared to sham. To control for the specificity of tACS effects on somatosensory mu-alpha oscillations, we additionally determined the effect of tACS on visual alpha oscillations. Therefore, on the basis of the ICA decomposed dataset of the pre-experiment we identified components that depicted visual alpha activity and thus had: (i) an amplitude peak in the alpha range of their amplitude spectra; (ii) had an occipital topography; and (iii) did not show any ERD to somatosensory stimulation. In analogy to the main analysis described above, the resulting components (number of selected components per subject: *Median* = 3; Range = 1–7) were used as spatial filters (see Figure 1C). Each participant’s individual visual alpha frequency was determined as the frequency with a maximum amplitude over occipital electrodes. Pre- and post-stimulation amplitudes measured at electrode POz were extracted and potential amplitude modulations by tACS or sham were tested as described above.

#### Debriefing

Difference between pre/post ratings of attention, vigilance, and pain were compared with a Wilcoxon Signed Ranks test. Data concerning the perception of the tACS stimulation onset, perception of differences in tACS and sham stimulation, and corresponding certainty ratings were analyzed quantitatively and qualitatively to examine whether subjects were able to identify tACS and sham blocks.

## Results

### Debriefing

Reported vigilance values (pre: median = 8, ranging from 7 to 10; post: median = 7, ranging from 5 to 10) decreased significantly during the course of the experiment (*Z* = −2.16; *p* = 0.031). Tiredness (pre: median = 3, ranging from 1 to 6; post: median = 4, ranging from 2 to 6) increased significantly (*Z* = 2.18; *p* = 0.029). Pain ratings (pre: median = 1, ranging from 1 to 2; post: median = 1, ranging from 1 to 2) however did not change significantly (*Z* = −1.00; *p* = 0.317).

Ten out of 23 participants reported that they felt the stimulation (burning, tingling, itching sensations) with a reported certainty of 10 (median = 10; range 3–10). However, participants did not systematically feel all stimulation blocks nor were they able to differentiate between verum and sham stimulation. Six participants reported to have felt one and four to have felt two blocks.

### Pre-Experiment

**Figure 2:**
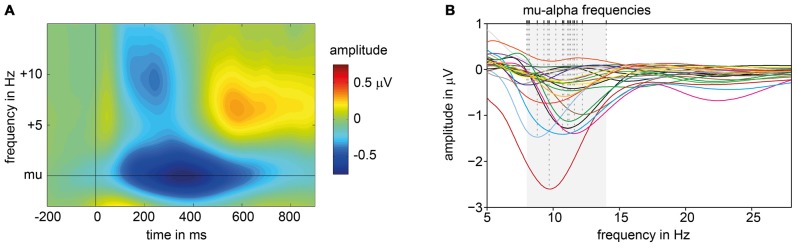
ERD patterns of pre-experiment. **(A)** Average, baseline corrected time frequency plot of signals measured at electrode C3 across all subjects, aligned to each participant’s individual mu-alpha frequency and presentation of suprathreshold electric stimuli (at 0 ms). **(B)** Subjects’ individual amplitude spectra for the time window averaged from 200 ms to 600 ms post-stimulus. Dashed lines mark individual mu-alpha peak frequencies with maximum ERD in alpha band (8–14 Hz, shaded in gray).

Based on the amplitude spectra of the signals measured at electrode C3 for 200–600 ms post stimulus, the peak frequency with the maximum ERD in the alpha range was extracted. ERD patterns varied in frequency (*M* = 10.608 Hz; *SD* = 1.496) and amplitude across subjects (see Figure 2B). When amplitude spectra are aligned to each subject’s individual mu-alpha-peak frequency a strong ERD pattern emerges. A decrease in amplitude is present for a time window of around 100–700 ms post-stimulus for the mu-alpha peak frequency and neighboring frequencies. Additionally a more transient decrease in the beta range (mu-alpha + around 10 Hz) is followed by a typical beta rebound (see Figure 2A).

### Main Experiment

**Figure 3:**
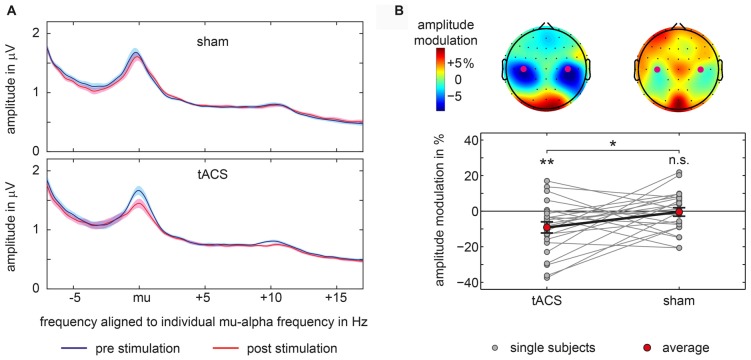
Amplitude modulations in main experiment. **(A)** Average amplitude spectra aligned to each participant’s individual mu-alpha frequency for sham and tACS-block separately for pre-stimulation and post-stimulation time window for signals measured above bilateral somatosensory cortices at electrodes C3 and C4. Shaded area represents 95% confidence intervals of the mean for within subject designs (Cousineau, 2005). **(B)** Topographical representations of mu-alpha amplitude modulations with electrodes C3 and C4 marked by purple dots are shown at the top. Bottom graph shows single subject and average pre to post-stimulation amplitude modulations of mu-alpha oscillations (mu-alpha ±1 Hz) measured above bilateral somatosensory cortices at electrodes C3 and C4. Significant differences from 0 or between conditions are marked with asterisks. **p < 0.01, *p < 0.05, n.s. *p* > 0.05.

Modulations of mu-alpha oscillations by tACS and sham were statistically compared. We found a significant difference between tACS- and sham-related modulation of mu-alpha oscillations (*t*_(22)_ = −2.134; *p* = 0.044; *d* = 0.445). As depicted in Figure 3B, there was a significant negative modulation of mu-alpha amplitude after the tACS block (*M* = −9.146; *SD* = 15.021; *t*_(22)_ = −2.920; *p* = 0.008; *d* = 0.609), localized bilaterally over central electrodes, while there was no modulation for sham stimulation (*M* = −0.452; *SD* = 11.500; *t*_(22)_ = −0.189; *p* = 0.852; *d* = 0.039). Importantly, pre-stimulation amplitude values for tACS and sham (tACS: *M* = 1.508; *SD* = 0.800; sham: *M* = 1.499; *SD* = 0.762; *t*_(22)_ = 0.131; *p* = 0.897; *d* = 0.027) were not different.

Moreover, as visible in Figure 3A this modulation was specific to the mu alpha range. Changes in amplitude seem not to be attributable to changes in participant’s peak frequency.

First, given, that the peaks in the amplitude spectra align to the individual mu-alpha frequency (see Figure 3A) there is no evidence for difference between ERD-based pre-experimental peak frequency and resting peak frequency. As a control measure, we extracted resting-state peak frequencies for each subject and directly compared them with the peak frequencies determined via ERD patterns in the pre-experiment. We found that mean pre-sham and pre-tACS peak frequencies (*M* = 10.160, *SD* = 1.213) were comparable with the ERD peak frequencies (see above) with no significant difference between them as revealed by a paired *t*-test: *t*_(22)_ = −0.218; *p* = 0.829; *d* = 0.046.

Second, there was no evidence for a systematic shift of the peak frequency throughout the experiment. We also extracted peak frequencies of each participant’s amplitude spectra for both time points (pre- vs. post-stimulation) as well as stimulation conditions (tACS vs. sham). Peak frequencies were comparable across conditions: tACS pre: *M* = 10.169, *SD* = 1.332; tACS post: *M* = 10.479, *SD* = 1.150; sham pre: *M* = 10.150, *SD* = 1.163; sham post: *M* = 10.233, *SD* = 1.151. A repeated measures ANOVA with the factors TIME and STIMULATION neither revealed any main effects nor an interaction: STIMULATION, *F*_(1,22)_ = 1.55, *p* = 0.227, *η*^2^ = 0.003; TIME, *F*_(1,22)_ = 1.89, *p* = 0.184, *η*^2^ = 0.007; STIMULATION × TIME, *F*_(1,22)_ = 0.56, *p* = 0.463, *η*^2^ = 0.002.

Mu-alpha peak frequencies seem therefore to be stable throughout the experiment.

**Figure 4:**
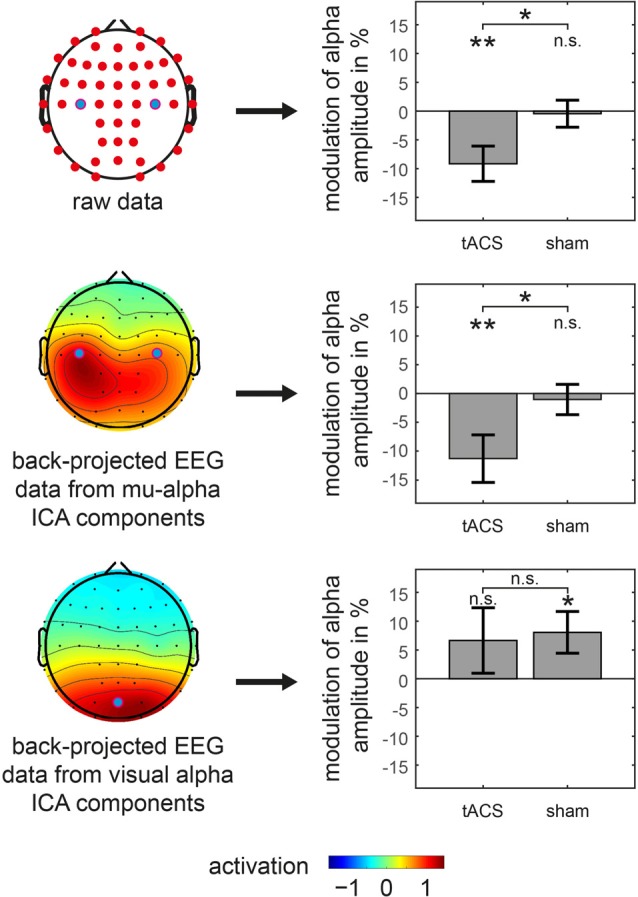
Amplitude modulations of specific oscillations in main experiment. tACS and sham related modulation of somatosensory mu-alpha amplitude (middle row) as well as visual alpha amplitude (bottom row) is shown. Analysis is based on back-projected EEG data of either mu-alpha or visual alpha ICA components. Topographies on the left represent average activation topographies of ICA matrices averaged across subjects (Note: scale has arbitrary units). Analyzed electrode channels are marked with blue dot. Bar graphs on the right show modulation of alpha amplitude with significant differences from 0 or between conditions marked with asterisks. **p < 0.01, *p < 0.05, n.s. *p* > 0.05. Error bars represent Standard Error. Results for analysis of raw EEG data is represented in top row for reference.

In order to test whether these findings are specific to the somatosensory mu-alpha target rhythm, in a second step, the analysis was constrained to signals from two different sources. First, based on an ICA-decomposition and the subsequent selection of specific somatosensory mu-alpha related components, signals were analyzed that specifically depicted somatosensory mu-alpha oscillations while suppressing signals of other alpha generators. Here the previously found modulation pattern was confirmed: a significant difference between the modulations of mu-alpha oscillations by tACS and sham was observable (*t*_(22)_ = −2.228; *p* = 0.036; *d* = 0.465) with a negative amplitude modulation by tACS (*M* = −11.293; *SD* = 20.147; *t*_(22)_ = −2.688; *p* = 0.013; *d* = 0.561) and no significant modulation by sham (*M* = −1.046; *SD* = 12.891; *t*_(22)_ = −0.389; *p* = 0.701; *d* = 0.081). There was no difference observable in pre-stimulation amplitudes (tACS: *M* = 1.129; *SD* = 0.834; sham: *M* = 1.120; *SD* = 0.822; *t*_(22)_ = 0.150; *p* = 0.882; *d* = 0.031; see Figure 4).

Moreover, when analyzing potential alterations of visual alpha oscillations, by constraining the analysis to EEG signals of visual alpha ICA components, there was no significant difference between the modulation of visual alpha oscillations by tACS and sham (*t*_(22)_ = −0.204; *p* = 0.840; *d* = 0.043) with no significant modulation for tACS (*M* = 6.644; *SD* = 27.897; *t*_(22)_ = 1.142; *p* = 0.266; *d* = 0.238) and a significant positive modulation for sham stimulation (*M* = 8.059; *SD* = 17.778; *t*_(22)_ = 2.174; *p* = 0.041; *d* = 0.453), while there were no significant pre-stimulation differences (tACS: *M* = 0.971; *SD* = 0.566; sham: *M* = 1.016; *SD* = 0.662; *t*_(22)_ = −1.126; *p* = 0.272; *d* = 0.235).

Taken together, after application of tACS but not sham, we observed a specific suppression of the amplitude of somatosensory mu-alpha but not visual alpha oscillations.

## Discussion

In the present study we investigated the effects of tACS, applied with individual mu-alpha peak frequencies bilaterally over both somatosensory cortices (mu-tACS), on ongoing somatosensory mu-alpha oscillations. In line with our hypothesis, mu-tACS specifically modulated somatosensory mu-alpha oscillations as evidenced by a decrease in mu alpha amplitude after stimulation, but did not modulate visual alpha oscillations.

Effects of tACS on ongoing neuronal oscillations in the human brain have as yet been shown in the visual domain only. When applying tACS at alpha frequency over visual cortex, increases in amplitude of alpha oscillations during and after stimulation were reported (Zaehle et al., 2010; Neuling et al., 2013; Helfrich et al., 2014b; Vossen et al., 2015; Kasten et al., 2016). The exact neurophysiological mechanisms underlying these increases in amplitude are still not fully understood. *In vivo* and *in vitro* studies in animal models found interactions between ongoing neural oscillatory activity and applied sinusoidal stimulation as potential basis for stimulation effects. The type of interaction is depending on parameters of amplitude of the applied stimulation and the relationship between frequencies of ongoing neural oscillations and applied oscillations (Reato et al., 2013). It has been suggested that applied electric oscillations may entrain ongoing neural oscillations leading to more coherent neural activity (Jefferys et al., 2003; Deans et al., 2007; Radman et al., 2007; Fröhlich and McCormick, 2010; Ozen et al., 2010; Reato et al., 2010). Due to resonance phenomena (Hutcheon and Yarom, 2000) applied sinusoidal currents may entrain ongoing neural oscillations most strongly when both frequencies match, while for differing frequencies entrainment requires higher intensity (Ozen et al., 2010; Ali et al., 2013; Merlet et al., 2013; Reato et al., 2013; Schmidt et al., 2014; Herrmann et al., 2016). Only recently, evidence supporting entrainment as mechanism of action underlying online-effects of tACS was reported in humans (Helfrich et al., 2014b; Neuling et al., 2015; Alagapan et al., 2016; Ruhnau et al., 2016). While such entrainment-related effects may account for amplitude modulations of neural oscillations during stimulation, effects outlasting the actual stimulation might be related to different mechanisms. For instance, outlasting effects were absent when using only short timed stimulation protocols of several seconds in animals (Deans et al., 2007) or in humans (Strüber et al., 2015) and were independent of a precise phase-coherent stimulation optimal for possible entrainment (Vossen et al., 2015). Based on this, it was proposed that plastic changes are responsible for tACS-induced offline effects, and may differ from online effects related to entrainment of neural oscillations by tACS (Zaehle et al., 2010; Strüber et al., 2015; Veniero et al., 2015; Vossen et al., 2015). Herrmann et al. (2013) suggested LTP-driven changes of synaptic weights to be a candidate mechanism for plastic changes. Accordingly, network activity that is coherently modulated by tACS with a frequency close to the network’s intrinsic frequency specifically strengthens the synaptic weights of dominant recurrent loops within this network responsible for the intrinsic oscillatory activity. Thus, the dominant oscillatory pattern in a neural network may be strengthened by tACS (Zaehle et al., 2010; Herrmann et al., 2013).

What may explain the disparity between our result and previous studies reporting an increase in amplitude after tACS? Here, differences in stimulation parameters as well as target brain areas and/or rhythms need to be considered and discussed. Our results might specifically relate to our target rhythm, somatosensory alpha, and the specific stimulation protocol used in our study. In case of visual alpha oscillations, neural generators usually show a centro-parieto-occipital distribution (Goldman et al., 2002; Klimesch et al., 2007; Mantini et al., 2007; van Dijk et al., 2008; Foxe and Snyder, 2011; Mathewson et al., 2011; Haegens et al., 2015) and were thus stimulated in phase either with a posterior-anterior or a left-right lateral placement of stimulation electrodes in previous tACS studies (Zaehle et al., 2010; Neuling et al., 2013; Helfrich et al., 2014b; Vossen et al., 2015; Kasten et al., 2016). Bilateral generators of mu-alpha oscillations on the other side are distributed much more laterally in primary left and right somatosensory cortices (Pfurtscheller et al., 1997; Ritter et al., 2009; Haegens et al., 2010, 2012; van Ede et al., 2011, 2014). With the bilateral electrode placement used in this study, current sinks under one electrode (e.g., in the vicinity of left mu-alpha generators) are mirrored by current peaks under the other (e.g., in the vicinity of right mu-alpha generators). Due to the cyclic change of current flow direction for alternating currents, generators of mu-alpha oscillations of both hemispheres are therefore stimulated antiphasically. This is in contrast to protocols reported for visual alpha oscillations, for which central alpha generators are mostly stimulated in phase with an anterior-posterior placement of electrodes (but see Zaehle et al., 2010). At the same time it is known that left and right somatosensory cortices are functionally coupled and may mutually co-modulate their neural activity (Manzoni et al., 1989; Blatow et al., 2007; Blankenburg et al., 2008; Wang et al., 2013). An antiphasic modulation of lateral mu-alpha generators by tACS with simultaneous processes of mutual excitation and inhibition may create dynamics in activity different from the in-phasic stimulation of visual alpha generators by tACS. This may lead to different stimulation outcomes for instance via metaplastic mechanisms towards homeostatic states (Abraham, 2008; Karabanov et al., 2015). In line with this proposition, a recent study using computer simulations of neural networks focused on the modulation of oscillatory network activity by electric alternating stimulation. Crucially, nodes comprising the networks were functionally coupled with propagation delays, resembling for instance the functional connections between both SI. Here, an in-phase stimulation of separate network nodes lead to stable increases in oscillatory activity throughout the network, which would even outlast the stimulation. For antiphasic stimulation, however, no increased oscillatory activity could be found (Kutchko and Fröhlich, 2013). Based on this, the outcome of oscillatory stimulation on functional networks may crucially depend on specific neuroanatomical properties and temporal dynamics of the stimulation (Esfahani et al., 2016). These questions could be examined by directly comparing stimulation protocols that either aim at modulating somatosensory cortex in a unilateral or in a bilateral fashion, which for direct current stimulation revealed different interhemispheric and intracortical stimulation effects (Lindenberg et al., 2013; Sehm et al., 2013).

As raised above, the timing of our effects in relation to the stimulation protocol might be of great importance. Previous findings demonstrated that mu-tACS lead to a phasic modulation of somatosensation (Gundlach et al., 2016) and tACS at various frequencies lead to a modulation of tactile sensations during stimulation with strongest effects for tACS in the alpha range (Feurra et al., 2011b). These results are well in line with the presumed mechanism of an entrainment of ongoing oscillations by tACS (Herrmann et al., 2013, 2016; Reato et al., 2013). However, as an online entrainment of oscillations is more likely to produce an increase in amplitude, the decrease in mu-alpha after stimulation points towards a different mechanism of tACS on network activity: in contrast to online-entrainment, the offline modulation of mu-alpha oscillations may indicate stimulation-induced (meta-)plastic processes (Abraham, 2008) in the sense of a homeostatic rebound of activity in the somatosensory network. Further investigation is needed to elucidate the nature of these potential mechanisms and their interactions.

In summary, we provide evidence that tACS applied at an endogenous frequency is capable of a specific modulation of the targeted somatosensory mu-alpha rhythm. The direction of our modulation with a decrease in mu-alpha might be related to homeostatic neuroplastic processes following the stimulation.

## Author Contributions

CG, BS, MMM and AV designed experiment. CG and BS conducted experiment. CG, BS and TN analyzed data. CG, BS, MMM, TN and AV wrote the manuscript.

## Conflict of Interest Statement

The authors declare that the research was conducted in the absence of any commercial or financial relationships that could be construed as a potential conflict of interest.
